# Chemical Constituents and Their Biological Activities from Genus *Styrax*

**DOI:** 10.3390/ph16071043

**Published:** 2023-07-22

**Authors:** Ding-Ding Xia, Xin-Yu Han, Yu Zhang, Na Zhang

**Affiliations:** 1 Shanghai Frontiers Science Center for Chinese Medicine Chemical Biology, Institute of Interdisciplinary Integrative Medicine Research, Shanghai University of Traditional Chinese Medicine, No. 1200, Cailun Road, Shanghai 201203, China; 2103084016@stu.htu.edu.cn (D.-D.X.); 1710201016@vip.henu.edu.cn (X.-Y.H.); 2 School of Chemistry and Chemical Engineering, Henan Normal University, 46 East of Construction Road, Xinxiang 453007, China; 3 Institute of Pharmacy, Pharmacy College of Henan University, Jinming District, Kaifeng 475004, China; 4 Department of Biology, Philipps University, Karl-von-Frisch-Straße 8, 35043 Marburg, Germany

**Keywords:** *Styrax* genus, biological activities, constituents, lignans, terpenoid

## Abstract

Plants from the genus *Styrax* have been extensively used in folk medicines to treat diseases such as skin diseases and peptic ulcers and as an antiseptic and analgesic. Most *Styrax* species, especially *Styrax tonkinensis*, which is used as an expectorant, antiseptic, and analgesic in Chinese traditional medicine, could screen resin after external injury. *Styrax* is also used in folk medicines in Korea to treat sore throat, bronchitis, cough, expectoration, paralysis, laryngitis, and inflammation. Different parts of various *Styrax* species can be widely employed for ethnopharmacological applications. Moreover, for ethnopharmacological use, these parts of *Styrax* species can be applied in combination with other folk medicines. *Styrax* species consist of versatile natural compounds, with some of them exhibiting particularly excellent pharmacological activities, such as cytotoxic, acetylcholinesterase inhibitory, antioxidant, and antifungal activities. Altogether, these exciting results indicate that a comprehensive review of plants belonging to this genus is essential for helping researchers to continuously conduct an in-depth investigation. In this review, the traditional uses, phytochemistry, corresponding pharmacological activities, and structure–activity relationships of different *Styrax* species are clarified and critically discussed. More insights into potential opportunities for future research are carefully assessed.

## 1. Introduction

The genus *Styrax* has a widespread but dispersive distribution. It is found in East Asian, American, and Mediterranean regions. It is the largest genus of the Styracaceae family and contains approximately 130 species [1]. *Styrax* stands out from other genera in this family because it produces a resinous material known as benzoin resin. This resin is typically released when the bark is injured by sharp objects. It has been utilized in various regions across the globe for its aromatic properties, being commonly used in perfumes and cosmetics, and *Styrax* species have traditionally been used in herbal medicines for the treatment of various diseases [2]. Of note, many *Styrax* species, especially *S. tonkinensis*, which is used as an expectorant, antiseptic, and analgesic in Chinese traditional medicine, could screen resin after exterior injury [3,4]. As a folk medicine in Korea, *S. japonica* is used to treat cough, bronchitis, sore throat, inflammation, paralysis, laryngitis, and expectoration [5,6,7]. The resin from *Styrax*, mixed with other antibiotic substances and hardening material, is also indicated in Islamic medicine as working as a good dental restorative material [2]. The flower of *S. japonicus* sieb. et Zucc. is used in Chinese folk medicine to relieve pain such as sore throat pain and toothache [8]. The leaves and roots of *Styrax suberifolium* are typically used as traditional medicines in China to cure rheumatic diseases [9].

**Figure 1:**
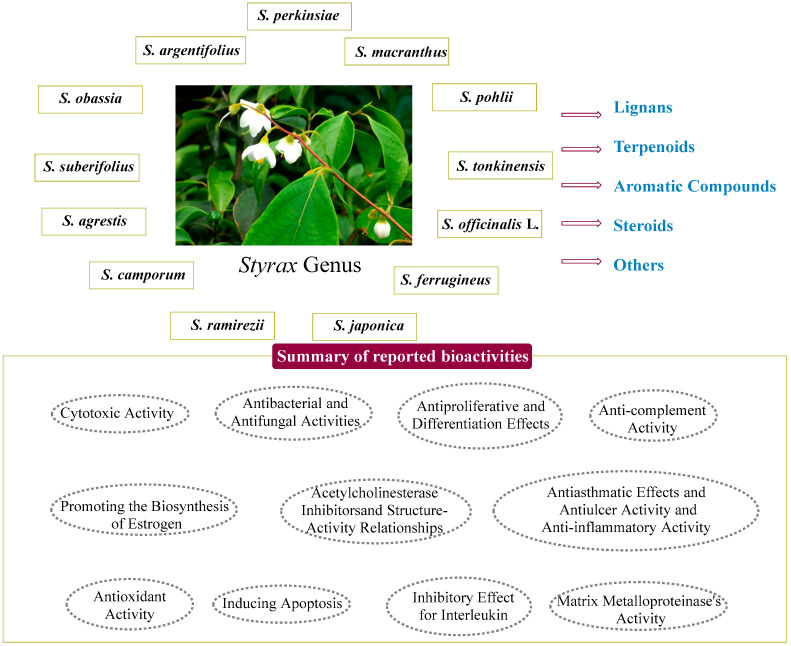
Research overview of the *Styrax* genus.

Furthermore, the extensive investigation of pharmacologically active compounds derived from various *Styrax* species has been ongoing for several decades. While numerous *Styrax* species have been studied, *S. obassia* and *S. japonica* have emerged as the most extensively researched species, encompassing studies ranging from phytochemistry to comprehensive pharmacological investigations (Figure 1). An example of the pharmacological potential of *Styrax* species was the inhibitory effect of benzofurans extracted from *Styrax agrestis* A. Chev. on acetylcholinesterase (AChE) in vitro [10]. Triterpenoids isolated from the resin of *S. tonkinensis* (Pier.) Craib showed promising antiproliferative and differentiation effects on human leukemia HL-60 cells [4]. Additionally, the hydroalcoholic extracts of *S. camporum* Pohl demonstrated effectiveness in reducing chromosome and DNA damage [11]. Another notable finding was the promotion of estrogen biosynthesis by egonol gentiobioside and egonol gentiotrioside from *Styrax perkinsiae* through the action of aromatase [12].

Despite significant progress in discovering natural compounds from *Styrax* species and elucidating their potential pharmacological activities, there is still a need for a comprehensive and focused discussion of this rapidly growing research area. With our continuous interests in natural products discovery and pharmacological research [13,14,15,16], our aim is to provide researchers with a convenient and comprehensive resource that offers detailed and concise profiles of the *Styrax* genus. This review encompasses the examination of structural diversity and the pharmacological and biological significance and presents the exciting future research prospects in this field.

## 2. Results and Discussion

### 2.1. Chemical Constituents

#### 2.1.1. Lignans

Lignans are the major constituents isolated from *Styrax* species. Most lignans are benzofuran, tetrahydrofuran, and furofuran lignans, and they are found in the stem nucleus of *S. perkinsiae*, *S. ferrugineus*, *S. macranthus*, *S. obassia*, *S. camporun*, *S. japonica*, and *S. officinalis* L. [2]. *S. perkinsiae* contains 16 norlignans including **1**–**14** and lignans **15** and **16** [17,18]. Norlignans (**2**, **4**, and **17**–**19**) from *S. ferrugineus* leaves were investigated and characterized [19]. Compounds **5** and **20**–**35** were isolated from the stem bark of *S. japonica* by several research groups [7,20,21,22,23,24]. Meanwhile, lignans **4**, **15**, and **16** were also afforded from *S. japonica* seeds [25]. Constituents of *S. obassia* were investigated, and norlignans (**2**, **3**, **6**, and **36**) were isolated [26]. Moreover, a series of reports revealed the presence of several benzofurans in *S. obassia* including **37**–**42** [27,28,29,30,31]. Six benzofuran derivatives comprising **4**–**7**, **14**, and **43**–**46** were afforded from the seeds of *S. macranthus* that grow in southwestern China [32,33]. Benzofurans **4**, **15**, **40**, and **47**–**49** were isolated from the hexane extract of the seeds of *S. officinalis* L. [34,35,36,37]. Thirteen compounds, **4**, **15**, **37**, **39**–**42**, and **50**–**55**, were obtained from the ethyl acetate (EtOAc) extract of the fruits of *S. agrestis* [10]. Moreover, compounds **4**, **17**, and **56** were isolated from *S. camporum*, and their protective activities were continuously assessed in vivo [11,38]. Bertanha et al. isolated benzofuran nor-neolignan derivatives **4**, **6**, **17**, **18**, and **57** from the aerial parts of *S. pohlii.* Several lignans including **58**–**65** were isolated from *S. perkinsiae* [39]. Seventeen phenylpropanoids were successfully isolated from the bark of *S. suberifolius*, including ten benzofuran derivatives (**4**–**5**, and **66**–**73**), two dihydrofuran derivatives (**23** and **65**), two new neolignans (**74** and **75**), and three benzalcohols (**76**–**78**) [40]. Eight lignans (**79**–**85**) were isolated from the leaves of *S. tonkinensis* (Pierre) Craib ex Hartw [41]. Two new phenylpropanoids (**86** and **87**) were isolated from the resin of *S. tonkinensis* (Pierre) Craib ex Hartw by Fang’s groups [42]. Two lignans (**88** and **89**) and five nor-lignan-type benzofurans, including **4** and **90**–**93**, were separated from *S. argentifolius* by Son’s group [43].

#### 2.1.2. Terpenoids

Terpenoids were also obtained from the *Styrax* genus as one of its major constituents. It should be noted that a vast majority of the terpenoids isolated from the *Styrax* genus were pentacyclic triterpenoids. To date, these molecules were only found in four species of the *Styrax* genus. Compounds **94**–**102** were isolated from the stem bark of *S. japonica* Sieb. et Zucc. by several research groups [20,22,44,45]. A phytochemical investigation on the fresh fruits of *S. japonica* Sieb. et Zucc. was also conducted, and four new triterpenoid glycosides including jegosaponins A–D (**103**–**106**) were found [46]. Furthermore, *S. japonica* Sieb. et Zucc. continued to be investigated by Kwon’s group, and **107**–**110** and taraxerol (**94**) were isolated [47]. In addition to the plants themselves, triterpenoids were also found from the resin of *S. tonkinensis* (Pier.) Craib containing **111**–**119** [4,6]. A pentacyclic triterpenoid (**120**), three triterpenoid saponins *styrax*-saponins A-C (**121**–**123**), and deacylsaponin (**124**) were also obtained from *S. officinalis* L. [48,49]. Moreover, several monoterpenes, such as *α*-terpineol, linalool, and geraniol, were isolated from the benzoe resin of *S. officinalis* L. [50]. Recently, two cinnamyl esters and seven pentacyclic triterpene acids (**119**, **125**–**130**) were separated and characterized from *S. tonkinensis* (Pierre) Craib ex Hartw [42,51]. A triterpenoid (**131**) was obtained from *S. argentifolius* very recently [43].

#### 2.1.3. Aromatic Compounds

Aromatic compounds, as a small proportion, were reported in the *Styrax* genus as well. In the species of *S. tonkinensis* (Pier.) Craib, seven aromatic compounds including **132**–**139** were reported [52,53]. Moreover, Kim and coworkers found **140** and **141** from the stem bark of *S. japonica* (SJ) [54]. In *S. perkinsiae* Rhed., **142** was separated [39]. Recently, a new epicatechin glucopyranoside, **143**, and three mononuclear phenolic acid esters, **144**–**146**, were isolated from the bark of *S. suberifolius* Hook [40].

#### 2.1.4. Steroids

Luo and coworkers reported that three steroids including stigmasterol (**147**), styraxosides A (**148**), and daucosterol (**149**) were obtained from the seeds of *S. macranthus* Perk [32]. Another Steroid named *β*-sitosterol (**150**) was reported in *S. perkinsiae Rehder* [17]. A sterol, **151**, was separated from *S. argentifolius* H.L. Li by Son’s group [43].

#### 2.1.5. Others

In addition to the commonly isolated products from the genus *Styrax*, other types of natural products were also reported with relatively limited numbers. For example, in 1973, a preliminary result regarding the seeds of *S. officinalis* L. showed that the oil content amounts to 50% [55]. Moreover, flavonoids are not frequently reported in the *Styrax* genus according to literature studies. Only four flavonoids including **152**–**155** were isolated from the aerial parts of *S. pohlii* A. DC. and the leaves of *S. camporum* Pohl [56]. Later, two new polyketones, **156**–**157**, were isolated from *Styrax camporum* Pohl. [57]. Recently, two bioactive saponins, Jegosaponin A and B (**158**–**159**), were extracted and subsequently identified from *S. japonica* Siebold et al. Zuccarini [58].

### 2.2. Chemical Constituents Biological Activities

#### 2.2.1. Cytotoxic Activity

**Figure 2:**
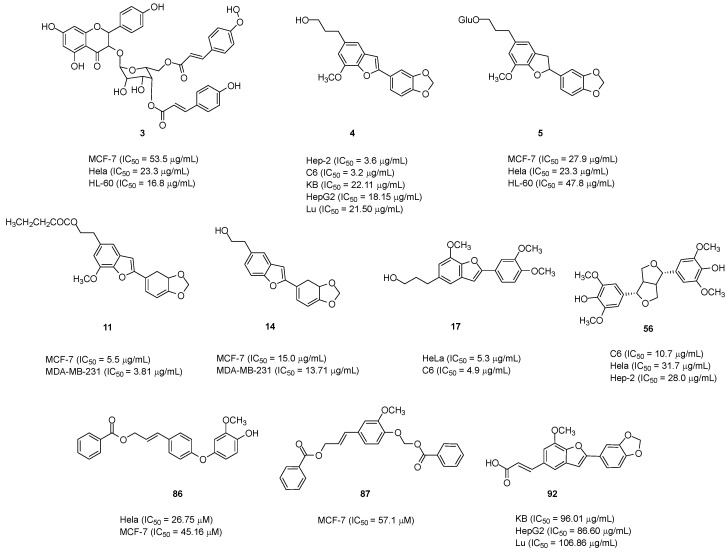
Natural compounds with cytotoxic activity.

*S. perkinsiae* was investigated, and the cytotoxic activity of the compounds isolated from this species was tested through the colorimetric chemosensitivity assay with SRB. (Figure 2). Interestingly, **11** and **14** revealed cytotoxic activities in vitro against two breast cancer cell lines, MCF-7 (IC_50_ = 5.5 and 15.0 µg/mL, respectively) and MDA-MB-231 (IC_50_ = 3.81 and 13.71 µg/mL, respectively) [17].

Later, the cytotoxic activities of lignans isolated from *S. camporum* against three cell lines, namely, HeLa (human cervix carcinoma), C6 (rat glioma), and Hep-2 (larynx epidermoid carcinoma), were analyzed using the standard MTT. Compound **4** showed strong cytotoxic activities against the Hep-2 (IC_50_ = 3.6 µg/mL) and C6 (IC_50_ = 3.2 µg/mL) cell lines. Compound **17** exhibited significant cytotoxic activities against the HeLa (IC_50_ = 5.3 µg/mL) and C6 (IC_50_ = 4.9 µg/mL) cell lines. Compound **56** exhibited moderate cytotoxic activities against the Hep-2 (IC_50_ = 28.0 µg/mL), HeLa (IC_50_ = 31.7 µg/mL), and C6 (IC_50_ = 10.7 µg/mL) cell lines. Moreover, when combined, **4** and **17** exhibited higher cytotoxic activities than the hydroalcoholic extract or either of the lignans alone, with the lowest IC_50_ being 13.3 µg/mL [38,59].

Seven compounds isolated from *S. obassia* were screened for their cytotoxic activities against the HeLa, HL-60, and MCF-7 cell lines. Among them, compounds **3** and **5** exhibited significant antitumor properties. Compound **3** exhibited cytotoxicity against the HeLa (IC_50_ = 23.3 µg/mL), HL-60 (IC_50_ = 16.8 µg/mL), and MCF-7 cells (IC_50_ = 53.5 µg/mL). Meanwhile, compound **5** exhibited cytotoxicity against HeLa (IC_50_ = 23.3 µg/mL), HL-60 (IC_50_ = 47.8 µg/mL), and MCF-7 cells (IC_50_ = 27.9 µg/mL) [60].

Through the Cell Counting Kit-8 (CCK-8) test in vitro, compounds **86** and **87** were tested for their cytotoxic activities against five tumor cell lines (PC-3, MCF-7, A549, HeLa, and HepG-2). Among them, the cytotoxic effect of compound **86** was observed against the MCF-7 and HeLa cell lines (IC_50_ = 26.75 and 45.16 µM, respectively), which was better or similar to that of the positive control cisplatin (IC_50_ = 40.95 and 47.36 μM, respectively). Compound **86** exhibited moderate cytotoxicity against the PC-3 and HepG-2 cell lines. The other biomolecule, **87**, displayed moderate cytotoxicity against MCF-7 cells (IC_50_ = 57.1 µM) [42].

Son’s group assessed the cytotoxicity and *α*-glucosidase inhibitory activity of isolated compounds from *S. argentifolius*. They suggested that the activities of triterpenoid **131** and norlignan-type benzofurans (**4** and **91**–**93**) are superior to those of others including sterol **153** and lignans **88** and **89**. The better activities of benzofurans (**4** and **91**–**93**) were postulated to be an effect of the substitutions at the side chain of carbon C-5. Among them, compound **4** exhibited potential cytotoxicity against three cancer cell lines, namely, Lu (IC_50_ = 21.50 µg/mL), KB (IC_50_ = 22.11 µg/mL), and HepG-2 (IC_50_ = 18.15 µg/mL) [43].

#### 2.2.2. Antibacterial and Antifungal Activity

**Figure 3:**
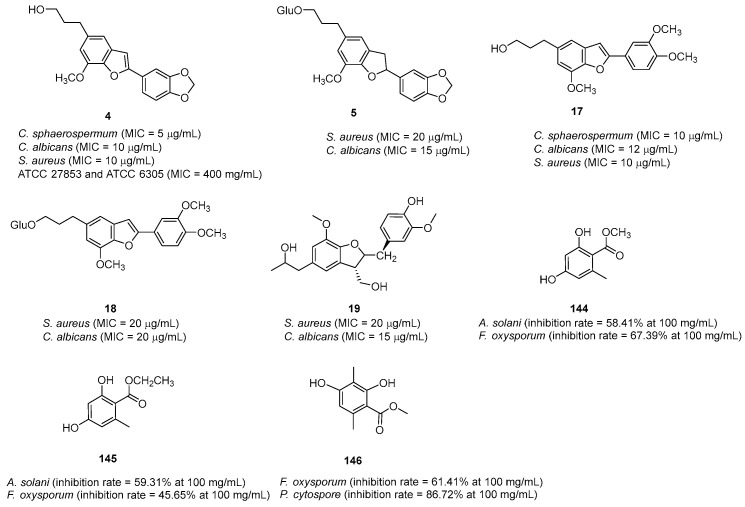
Natural compounds with antibacterial and antifungal activities.

Initially, the extract of *S. ferrugineus* exhibited antifungal and antibacterial activities against *Candida albicans*, *Cladosprorium sphaerospermum*, and *Staphylococcus aureus*. To identify the potential biomolecules from this species that exhibit antifungal and antibacterial activities, the isolated lignans were tested (Figure 3). Among them, lignans **4** and **17** exhibited antifungal and antibacterial activities against *S. aureus* (MIC = 10 μg/mL and 20 μg/mL, respectively), *C. albicans* (MIC = 10 μg/mL and 12 μg/mL, respectively), and *C. sphaerospermum* (MIC = 5 μg/mL and 10 μg/mL, respectively), whereas the other three natural products (**5**, **18**, and **19**) only inhibited *C*. *albicans* (MIC = 15 μg/mL, 20 μg/mL, and 15 μg/mL, respectively) and *S. aureus* (MIC = 20 μg/mL, 20 μg/mL, and 20 μg/mL, respectively) [19].

To exploit the antibacterial activity of the aerial parts of *S. pohlii*, different fractions, especially those extracted using *n*-hexane, EtOAc, *n*-BuOH, and methanol, were evaluated against Haemophilus influenzae, Pseudomonas aeruginosa, *S. pyogenes*, *Streptococcus pneumoniae*, and *Klebsiella pneumoniae*. The broth microdilution method was used for measuring the minimum inhibitory concentration (MIC). Among the fractions, the n-hexane fraction exhibited excellent antibacterial activity against Gram-positive *S. pneumoniae* (MIC = 200 μg/mL). The MIC values of compounds **4** and **17** (400.0 µg/mL) against *P. aeruginosa* and *S. pneumoniae* were the best [61]. 

By conducting the radial growth-inhibition experiment, the antifungal activities of compounds from the bark of *S. suberifolius* against three plants’ fungal pathogen, namely, Phomopsis cytospore, Fusarium oxysporum, and Alternaria Solani, was exhibited. Compounds **144**, **145**, and **146** exhibited selective suppressive activities against the tested fungi. Notably, compound 146 was a significantly effective inhibitor of Phomopsis cytospore at 100.0 µg/mL, with an inhibition rate of 86.72% [40].

#### 2.2.3. Antiproliferative and Differentiation Effects

**Figure 4:**
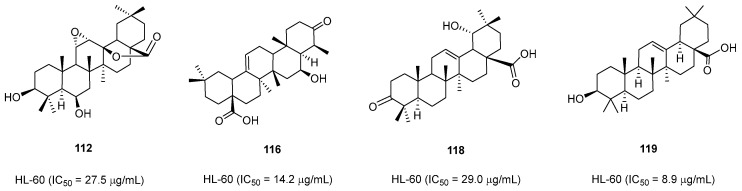
Natural compounds with antiproliferative and differentiation properties.

In 2006, Wang’s group found that triterpenoids (**111**–**120**) isolated from *S. tonkinensis* inhibit HL-60 cell growth (IC_50_ = 8.9–99.4 µM). Of note, oleanolic acid **119** acted as the most effective antiproliferative agent (IC_50_ = 8.9 µg/mL) (Figure 4). Compound **113** exhibited the lowest growth-inhibitory effect. According to the NBT-reduction assay, compound **113** induced HL-60 cell differentiation, as measured in [4].

#### 2.2.4. Anti-Complement Activity

**Figure 5:**
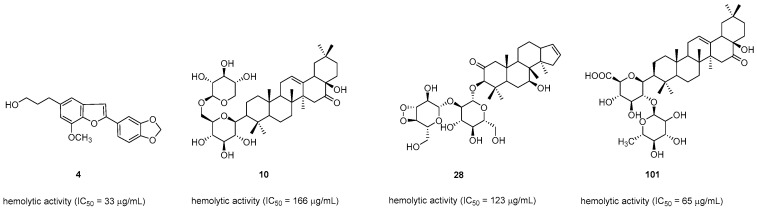
Natural compounds with anti-complement activity.

Egonol (**4**), masutakeside I (**10**), styraxlignolide A (**28**), and styraxoside B (**101**) isolated from *S. japonica* could inhibit the hemolytic activity of the complement system (IC_50_ = 33, 166, 123, and 65 µM, respectively) (Figure 5). This finding strongly suggested that the methyl enedioxy group of lignans has a vital role in inhibiting the hemolytic activity of human serum against erythrocytes [22]. 

#### 2.2.5. Anti-Complement Activity

**Figure 6:**
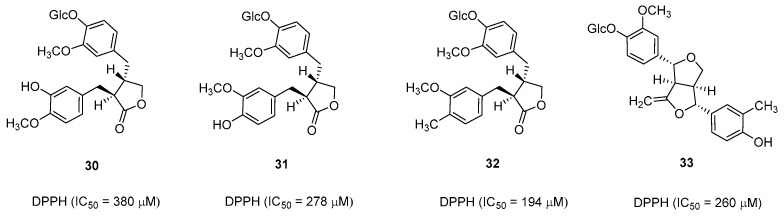
Natural compounds with antioxidant activity.

Natural products isolated from *S. japonica* were tested for in vitro antioxidant activities through the DPPH radical scavenging test. Among them, **30**–**33** exhibited weak DPPH radical scavenging activities (IC_50_ = 380, 278, 194, and 260 µM, respectively) (Figure 6) [7]. Moreover, Oliveira et al. reported that the hydroalcoholic extract of *S. camporum* could concentration-dependently scavenge DPPH radicals; a maximum scavenging activity of 85% was observed at 30.0 µg/mL [11].

#### 2.2.6. Induction of Apoptosis

Lee and Lim revealed that the ethanol extract of *S. japonica* Siebold et al., Zuccarini (SJSZ) induced programmed cell death (apoptosis) in HepG2 cells under the experimental condition (75.0 µg/mL of SJSZ for 4 h treatment). The results indicated that the ethanol extract of SJSZ (75 µg/mL) stimulates an increase in the number of iROS, Ca^2+^, and the apoptotic-related factors in HepG2 cells [62].

#### 2.2.7. Induction of Apoptosis

**Figure 7:**
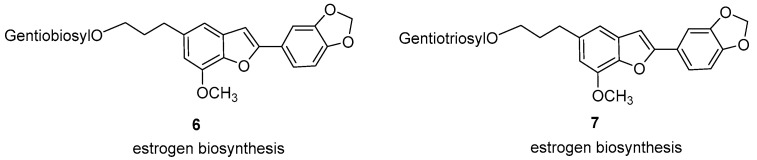
Natural compounds exhibiting estrogen-promoting activity.

In 2002, a nonradioactive assay was established for measuring aromatase activity by using human ovarian granulosa KGN cells. Lignans **6** and **7** exhibited approximately 1.62- and 1.95-fold increases, respectively, in 17 *β*-estradiol biosynthesis at 10 µM, and significantly improved 17 *β*-estradiol biosynthesis by approximately 1.53- and 1.71-fold, respectively, in 3T3-L1 preadipocyte cells (Figure 7). Moreover, egonol gentiotrioside increased serum estrogen levels in ovariectomized rats. These results suggested that these two lignans induce estrogen biosynthesis through the allosteric regulation of aromatase activity [12].

#### 2.2.8. Acetylcholinesterase Inhibitors and Structure–Activity Relationships

**Figure 8:**
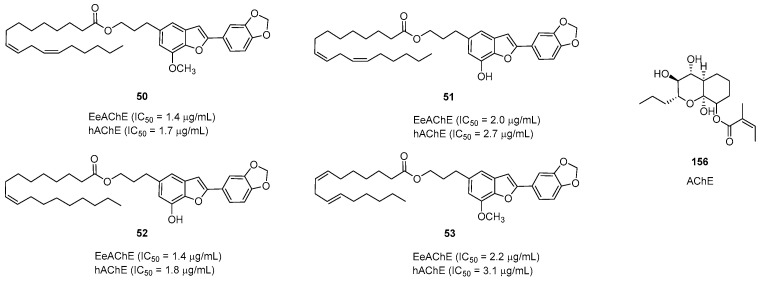
Natural compounds as potential acetylcholinesterase inhibitors.

In 2011, Liu et al. screened their library of plant extracts through a high-throughput assay. They found that the EtOAc extract of *S. agrestis* fruits exhibited significant inhibitory activity against AChE. They proved that two active subfractions were responsible for this inhibition and further isolated 13 compounds from the EtOAc extract. Later, they examined the selectivity and inhibitory potency of benzofurans on *h*AChE, BChE, and E*e*AChE by using the improved Ellman’s colorimetric method (Figure 8). Some egonol derivatives were synthesized through chemical modifications to clearly understand the structure–activity relationships. According to the results, the inhibition ratio affects the bulkiness and length of the alkyl ester group. In particular, compounds **50**–**53** exhibited inhibitory activity against AChE (IC_50_ = 1.4–3.1 μM). Compound **50** at 100.0 μM displayed obvious inhibition of A*β* aggregation (77.6%). Liu et al.’s SAR (Structure-Activity Relationships) studies indicated that compounds exhibiting anti-AChE activity are observed with the incorporation of alkyl chains consisting of more than three carbon units, the furan ring, and the ester group. Molecular docking studies proposed a binding site for this class of compound on AChE and identified multiple key residues at the peripheral site that are crucial for mediating the inhibitory effect [10]. The anti-AChE and antifungal activities of two novel polyketides, **156** and **157**, were also tested through TLC bioautographic assays. The results indicated that compound **156** could inhibit AChE activity [57]. 

#### 2.2.9. Inhibitory Effect on Interleukin

Lee and Lim separated a glycoprotein with an approximate molecular mass of 38 kDa from *S. japonica*. Subsequently, an immunoblot analysis and RT-PCR were conducted to evaluate ERK, JNK, and NF-*κ*B activities and the levels of inflammation-related factors (COX-2, inducible nitric oxide synthase (iNOS), and interleukin (IL)-1*β*) in Cr-induced BNL CL.2 cells. The SJSZ glycoprotein (50.0 µg/mL) inhibited the expression of ERK, NF-*κ*B, JNK, iNOS, IL-1*β*, and COX-2 [63].

With further investigation of the SJSZ glycoprotein (38 kDa), Lee and Kim proved that this glycoprotein modulates IFN-*γ*, IL-2, and IL-12 expression in cyclophosphamide (CTX)-induced Balb/c mice. The glycoprotein counteracted the CTX-induced immunosuppressive effects. It effectively restored the spleen and thymus weights to normal levels and enhanced the phagocytic activity of peritoneal macrophages in response to CTX. Furthermore, the SJSZ glycoprotein exerted regulatory effects on the proliferation of T and B lymphocytes, cytotoxicity of NK cells, and production of key cytokines (IIFN-*γ*, L-2, and IL-12). Additionally, it improved the activity of antioxidant enzymes (e.g., SOD, CAT, and GPx) [64].

#### 2.2.10. Matrix Metalloproteinase’s Activity

**Figure 9:**
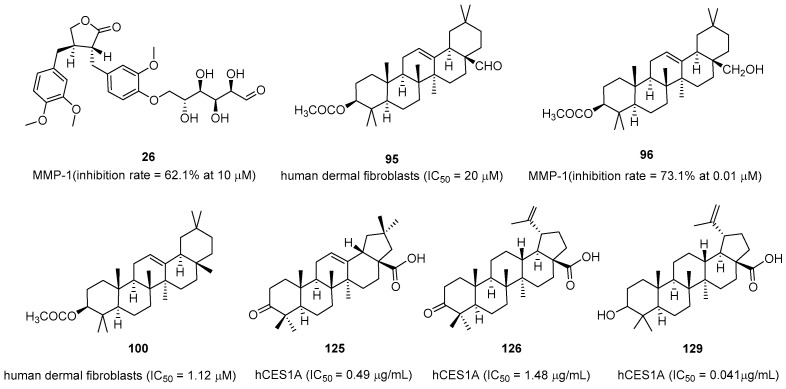
Natural compounds with matrix metalloproteinase’s activity.

Some triterpenoids (**95**–**97**, **100**) were isolated and further tested the Matrix Metalloproteinases (MMPs)’ activity of the methylene chloride soluble fraction of a methanol extract from the stems of *S. japonica.* Among them, **95** and **100** displayed effective cytotoxic activities against human dermal fibroblasts (IC_50_ = 20.0 and 1.12 µM, respectively) (Figure 9). In addition, **96** and **97** exhibited no cytotoxicity for the same cells at the test dose (0.01–1 µM). However, **96** dose-dependently reduced UV-induced MMP-1 protein levels to normal levels by 73.1% at 0.01 µM [65]. In a dose-dependent manner, **96** effectively downregulated MMP-1 protein expression, whereas it upregulated type-1 procollagen protein expression in the UV-irradiated cultured human skin fibroblasts of an elderly person [66].

*Styrax* japonoside B (**26**) exerted inhibitory activity against MMP-1 and prevented UV-induced changes in MMP-1 expression. At 10 µM, the treatment led to a significant dose-dependent reduction in MMP-1 protein expression, with an average decrease of 62.1% compared with the vehicle-treated control cells. The findings suggested that the glycoprotein can potentially be used as a potent antimetastatic agent. This glycoprotein exerts its effect by suppressing MMP-9 enzymatic activity through the NF-*κ*B and AP-1 signaling pathways [67].

Two cinnamyl esters (**86** and **87**) and seven pentacyclic triterpene acids (**119** and **125**–**130**) in *Styrax* are the key components that inhibit hCES1A activity. These seven pentacyclic triterpene acids in the two active sites of *Styrax* exert a significant inhibitory effect on hCES1A (IC_50_ = 41–478 nM). Among them, epibetulinic acid (**129**) (IC_50_ = 0.041 µg/mL), oleanonic acid (**125**) (IC_50_ = 0.49 µg/mL), and betulonic acid (**126**) (IC_50_ = 1.48 µg/mL) exhibited the strongest inhibitory activity against hCES1A [42,51].

#### 2.2.11. Antiasthmatic, Antiulcer, and Anti-Inflammatory Activities

**Figure 10:**
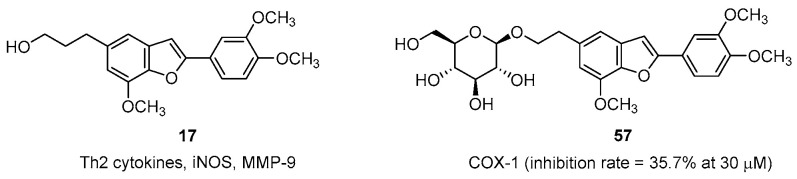
Natural compounds with antiasthmatic, antiulcer, and anti-inflammatory activities.The EtOAc fraction of *S. camporum* displayed antiulcer activity. It reduced the ulcer area and gastric secretion volume and increased the number of collagen fibers [37,68].

In a murine asthma model, homoegonol (**17**) exerted significant effects in reducing inflammatory cell infiltration and Th2 cytokine production in the bronchoalveolar lavage fluid. It also attenuated airway hyperresponsiveness, decreased serum IgE levels, and downregulated iNOS and MMP-9 expression. Thus, compound **17** exhibited the potential to effectively suppress OVA challenge-induced asthmatic responses (Figure 10).

In 2005, the extracted fractions of *S. pohlii* aerial parts, including the EtOAc fraction, ethanolic extract, and hexane fraction, were evaluated for their inhibitory activities against COX-1 and COX-2. The isolated products were further assessed against COX-1 and COX-2. The results revealed that all crude fractions and isolated products induced weak-to-moderate COX-1 and COX-2 inhibition. Among them, **57** exerted mild COX-1 inhibition, of 35.7% at 30 µM [69].

#### 2.2.12. Other Activity

Through micronucleus and comet assays, Oliveira demonstrated that different doses (250, 500, and 1000 mg/kg body weight) of the *S. camporum* extract’s compounds **4** and **17** had no genotoxic effect in Swiss mice. Moreover, they were effective in reducing doxorubicin- and methanesulfonate-induced DNA and chromosomal damage [11].

Braguine [69] investigated the EtOAc fractions of *S. camporum* and *S. pohlii* and isolated and identified compounds **152**–**155**. Upon biological evaluation, they found that the EtOAc fractions, as well as compounds **152** and **155**, could separate coupled Schistosoma mansoni adult worms. Additionally, compound **155** killed adult schistosomes in vitro. This research group also observed that homoegonol and homoegonol glucoside exhibited the best results against *S. mansoni* adult worms [70].

In vitro assessments were conducted to determine the protein tyrosine phosphatase 1B (PTP1B)’s inhibitory activities of compounds from *S. japonica* stem bark. Among the isolated compounds, **108** and **109** had the highest inhibitory activities (IC_50_ = 7.8 and 9.3 μM, respectively) [45].

By downregulating NF-*κ*B–DNA binding activity, styraxoside A (**148**) derived from *S. japonica* exerted inhibitory effects on the expression of LPS-induced iNOS, COX-2, tumor necrosis factor-*α*, and IL-1*β* [45].

**Figure 11:**
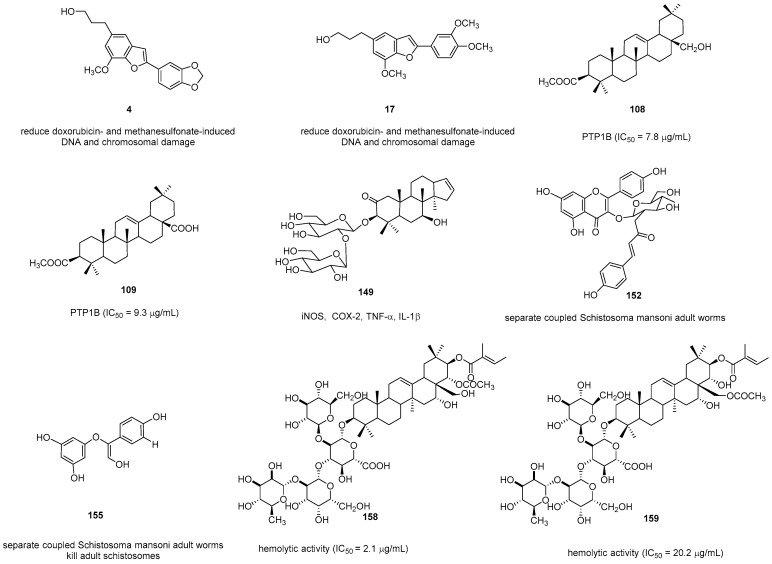
Natural compounds with other properties.

Jegosaponins A and B (**158** and **159**, respectively) exhibited potent hemolytic activity in sheep defibrillation (IC_50_ = 2.1 and 20.2 µg/mL, respectively) and could improve the performance of PC-3 cells and zebrafish embryos through the identification of a membrane nonpermeable DRAQ7, which is a fluorescent nucleus staining dye [58] (Figure 11).

## 3. Materials and Methods

Through the search of a variety of online libraries such as Wiley Online Library, PubMed, Scifinder Web, ACS, and Web of Science, a summary of the newly discovered chemicals isolated from the genus *styrax* and their related biological activities in recent decades was provided. All species names were checked using http://www.theplantlist.org (accessed on 10 May 2023).

## 4. Conclusions

In summary, the *Styrax* genus comprises 130 species, and most of the species are extensively used as traditional medicines (Appendix A), particularly in China and Korea. *Styrax* can be easily collected because of its extensive distribution. All the species of the Styrax family, which were reported regarding the aspects of phytochemistry and pharmacology, were comprehensively summarized. In total, 159 compounds (Appendix B), including lignans, terpenoids, steroids, etc., were isolated from various species. The biological activities of those isolated compounds were subsequently investigated, exhibiting broad bioactivities such as cytotoxic activity, antioxidant activity, antifungal activity, apoptotic activity, anti-inflammation activity, anti-complement activity and so on. Chemical and pharmacological studies on the *Styrax* genus also proved that its main constituents are lignans and terpenoids. Moreover, several bioactive molecules exhibiting strong pharmacological activities were also isolated from *Styrax* (Appendix C).

Of note, information about the structure–activity relationships of most bioactive compounds is insufficient due to the lack of derivatives. Therefore, the exploitation of the versatility of the potentially bioactive natural compounds obtained from this genus is in great demand. Moreover, some species used in traditional medicines are still untapped such as *S. suberifolius*, which is used as a cure for rheumatic arthritis, whereas the modern physiochemical and pharmacological investigations are missing. Furthermore, in-depth pharmacological studies, especially in vivo studies, of the isolated biomolecules should be conducted in the future.

## Data Availability

Not applicable.

## Abbreviations

MCF-7	Metastatic breast adenocarcinoma cell line
MDA-MB-231	Human breast cancer cell line
Hep-2	Human larynx carcinoma cell line
HeLa	Human cervix carcinoma cell lines
C6	Rat glioma cell lines
HL-60	Human leukemia cell line
A549	Human lung cancer cell line
PC-3	Human prostatic cancer cell line
Lu	Lung cancer cell lines
CCK-8	Cell Counting Kit-8
ATCC 6305	Streptococcus pneumoniae
ATCC 19615	Streptococcus pyogenes
ATCC 10211	Hemophilus influenzae
ATCC 27853	Pseudomonas aeruginosa
ATCC 10031	Klebsiella pneumoniae
MIC	Minimum inhibitory concentration
DPPH	DPPH radical
HepG2	Hepatocellular carcinoma cell line
KGN	Human ovarian granulosa cells
AChE	Acetylcholinesterase
E*e*AChE	Electrophorus electricus AChE
*h*AChE	Human AChE
BChE	Butyrylcholinesterase
CTX	Cyclophosphamide
IFN-*γ*	Interferon-*γ*
hCES1A	Human carboxylesterase 1A1
MMP-1	Matrix metalloproteinase-1
PTP1B	The protein tyrosine phosphatase 1B
iNOS	Nitric oxide synthase
TNF-*α*	Tumor necrosis factor-*α*
IL-1*β*	Interleukin-1*β*
COX-1	Cyclooxygenase-1
COX-2	Cyclooxygenase-2
NF-*κ*b	Nuclear factor-*κ*B

## Appendix A

**Table A1:** Genus *Styrax* and their traditional use.

Genus *Styrax*	Traditional Use
*S. perkinsiae*	Estrogen synthesis promotion
*S. obassia*	Anticancer activities
*S. japonica*	Cough, bronchitis, sore throat, inflammation, paralysis, laryngitis, and expectoration treatment
*S. pohlii*	/
*S. camporum*	Antimicrobial, anticancer, antifungal, hypolipidemic, and immunossupressive activities
*S. macranthus*	/
*S. officinalis*	Antiseptic and anti-respiratory disease
*S. argentifolius*	/
*S. ferrugineus*	/
*S. agrestis*	/
*S. tonkinensis*	Expectorant, antiseptic, and analgesic activities
*S. suberifolius*	Rheumatic disease cure
*S. ramirezii*	/

## Appendix B

**Table A2:** Chemical constituents of plants from the genus *Styrax*.

No.	Compound Class and Name	Source	Ref.
**Lignans**			
**1**	5-(2-Propen-1-one)-7-me-thoxy-2-(3,4-methylenedioxyphenyl) benzofuran	*S. perkinsiae*	[18]
**2**	1″-Hydryoxyegonol gentiobioside	*S. perkinsiae*	[18]
		*S. obassia*	[2]
		*S. ferrugineus*	[19]
**3**	Obassioside B	*S. perkinsiae*	[18]
		*S. obassia*	[2]
		*S. obassia* *S. obassia*	[2,60]
**4**	Egonol	*S. perkinsiae*	[18]
		*S. japonica*	[21,25]
		*S. obassia*	[28,31]
		*S. agrestis*	[10]
		*S. ferrugineus*	[19]
		*S. pohlii*	[69]
		*S. camporum*	[11]
		*S. macranthus*	[32]
		*S. officinalis* L.	[35]
		*S. argentifolius*	[43]
**5**	Egonol glucoside	*S. perkinsiae*	[18]
		*S. japonica*	[20]
		*S. suberifolius*	[40]
		*S. macranthus*	[32]
		*S. obassia*	[28]
			[60]
**6**	Egonol gentiobioside	*S. perkinsiae*	[17,18]
		*S. macranthus*	[32]
		*S. obassia*	[2]
		*S. pohlii*	[69]
**7**	Egonol gentiotrioside	*S. perkinsiae*	[17,18]
		*S. macranthus*	[32]
**8**	MasutakesideI (sutakeside I)	*S. perkinsiae*	[18]
		*S. japonica*	[22]
		*S. obassia*	[27]
**9**	*trans*-5-(3-Hydroxypropyl)-7-methoxy-2-[3-methoxy-4-hydroxyphenyl)-benzofuran-5-yl]benzufuran	*S. perkinsiae*	[17]
**10**	(*E*)-5-(2-Formylvinyl)-7-metho-xy-2-(3,4-methylenedioxyphenyl)benzofuran	*S. perkinsiae*	[17]
**11**	5-(3-Butanoyloxypropyl)-7-methoxy-2-(3,4-methylene dioxyphenyl)benzofuran	*S. perkinsiae*	[17]
**12**	5-(3-Hydroxypropyl)-7-hydroxy-2-(3,4-methylene-dioxyphenyl) benzofuran	*S. perkinsiae*	[17]
		*S. macranthus*	[32]
**12**	5-(3-Hydroxypropyl)-7-hydroxy-2-(3,4-methylene-dioxyphenyl) benzofuran	*S. perkinsiae*	[17]
		*S. macranthus*	[32]
**13**	Egonol acetate	*S. perkinsiae*	[17]
		*S. japonica*	[25]
		*S. obassia*	[28,29,31]
		*S. agrestis*	[10]
		*S. officinalis* L.	[37]
**14**	Demethoxy egonol acetate	*S. perkinsiae*	[17]
		*S. japonica*	[25]
**15**	Styraxlignolide B	*S. perkinsiae*	[18]
		*S. japonica*	[7]
**16**	Styraxjaponoside C	*S. perkinsiae*	[18]
		*S. japonica*	[7]
**17**	Nor-lignans5-(3″-hydroxypropyl)-7-methoxy-2-(3′,4′-methylenedioxyphenyl) benzofuran	*S. ferrugineus*	[19]
		*S. camporum*	[11]
		*S. pohlii*	[69]
**18**	5-[3″-(*β*-*D*-Glucopyranosyloxy)propyl]-7-methoxy-2-(3′,4′-methylenedioxyphenyl) benzofuran	*S. ferrugineus*	[19]
		*S. pohlii*	[69]
**19**	Dihydrodehydrodiconiferyl alcohol	*S. ferrugineus*	[19]
**20**	Styraxjaponoside C	*S. japonica*	[21]
**21**	Arctiin	*S. japonica*	[21]
**22**	Matairesinoside	*S. japonica*	[20,21]
**23**	Pinoresinol-4-*O*-*β*-*D*-glucopyranoside	*S. japonica*	[21]
**24**	2*R*-(4′-hydroxy-3′-methoxyphenyl)-6R-(3″,4″-methylenedioxyphenyl)-8-*oxo*-3,7-dioxabicyclo [3.3.0]octane 4′-hydroxyl	*S. japonica*	[7]
**25**	Styraxjaponoside A	*S. japonica*	[20]
**26**	Styraxjaponoside B	*S. japonica*	[20]
**27**	Dihydrodehydrodiconiferyl alcohol 9-*O*-glucoside	*S. japonica*	[20]
**28**	Styraxlignolide A	*S. japonica*	[22]
**29**	Styraxlignolide B	*S. japonica*	[7]
**30**	Styraxlignolide C	*S. japonica*	[7]
**31**	Styraxlignolide D	*S. japonica*	[7]
**32**	Styrax lignolides F	*S. japonica*	[7,24]
**33**	(–)-Pinoresinol glucoside	*S. japonica*	[7]
**34**	Styrlignan A	*S. japonica*	[23]
**35**	1*R*,2*R*,5*S*,6*R*-2-(4′-Hydroxy-3′-methoxyphenyl)-6-(3″,4″-dimethoxyphenyl)-3,7-dioxabicyclo-[3.3.0]octane4′-*O*-*β*-*D*-glucopyranoside	*S. japonica*	[24]
**36**	Obassioside A	*S. japonica*	[26]
**37**	Methyl 3-[7-methoxy-2-(3′,4′-methylene-dioxyphenyl)-5-benzofuranyl]-propionate	*S. obassia*	[31]
**38**	Methyl3-[2-(3′,4′-methylen-edioxyphenyl)-5-benzofuranyl]-propionate	*S. obassia*	[31]
**39**	5-(3″-Propanoyloxypropyl)-7-methoxy-2-(3′,4′-methylenedioxyphenyl)-benzofuran	*S. obassia*	[28]
		*S. agrestis*	[10]
**40**	Egonol-2-methylbutanoate	*S. obassia*	[31]
**41**	7-Demethoxylegonol-2-methylbutanoate	*S. obassia*	[5]
**42**	Egonol propanoate	*S. obassia*	[29]
		*S. agrestis*	[10]
**43**	3-[7-Methoxy-2-(3,4-methylenedioxyphenyl)benzofuran-5-yl]propyl-3-[7-methoxy-2-(3,4-methylenedioxyphenyl)-benzofuran-5-yl]propanoate	*S. macranthus*	[32]
**44**	Demethoxy egonol gentiobioside	*S. macranthus*	[32]
**45**	7-Methoxy-2-(3,4-methylenedioxyphenyl) benzofuran-5-carbaldehyde	*S. macranthus*	[32]
**46**	Demethoxy egonol	*S. macranthus*	[32]
		*S. obassia*	[28]
		*S. japonica*	[23]
**47**	5-3″-(2-Methylbutanoyloxy)propyl]-7-methoxy-2-(3′,4′-dimethoxyphenyl) benzofuran	*S. officinalis* L.	[23]
**48**	5-(3″Benzoyloxypropyl)-7-methoxy-2-(3′,4′-methylenedioxyphenyl)-benzofuran	*S. officinalis* L.	[36]
**49**	4-[3″-(1c-methylbutanoyloxy)propyl]-2-methoxy-(3′,4′-methylenedioxyphenyl)-1a,5b-dihydrobenzo-[3,4]-cyclobutaoxirene	*S. officinalis* L.	[65]
**50**	Egonol-9(Z),12(Z) linoleate	*S. agrestis*	[10]
**51**	7-Demethoxyegonol-9(Z),12(Z) linoleate	*S. agrestis*	[10]
**52**	7-Demethoxyegonol oleate	*S. agrestis*	[10]
**53**	Egonol oleate	*S. agrestis*	[10]
**54**	7-Demethoxylegonol acetate	*S. agrestis*	[10]
**55**	Egonol-2-methylpropanoat	*S. agrestis*	[10]
**56**	(±)Syringaresinol	*S. camporum*	[38]
**57**	Homoegonol gentiobioside	*S. pohlii*	[39]
**58**	Lariciresinol 4-*O*-*β*-*D*-glucoside	*S. perkinsiae*	[39]
**59**	(−)-Secoisolariciresinol 4-O-*β*-*D*-Glucopyranoside	*S. perkinsiae*	[39]
**60**	Lariciresinol4′-*O*-*β*-*D*-glucoside	*S. perkinsiae*	[39]
**61**	Lanicepside A	*S. perkinsiae*	[39]
**62**	Solariciresinol4-*O*-*β*-*D*-glucopyranoside	*S. perkinsiae*	[39]
**63**	(+)-Lariciresinol9-*O*-*β*-*D*-glucopyranoside	*S. perkinsiae*	[39]
**64**	2*R*,3*S*-Dihydrodehydrodiconiferyl alcohol 4′-*O*-*β*-*D*-glucopyranoside	*S. perkinsiae*	[39]
**65**	Pinoresinol	*S. perkinsiae*	[39]
**66**	Homoegonol glucoside	*S. suberifolius*	[40]
**67**	2-(4-Hydroxy3-methoxyphenyl)-5-(3-hydroxypropyl)-7-methoxybenzofuran	*S. suberifolius*	[40]
**68**	2-(3-Hydroxy-4-methoxyphenyl)-7-methoxy-5benzofuranpropanol	*S. suberifolius*	[40]
**69**	(+)-Cedrusin	*S. suberifolius*	[40]
**70**	(−)-(7R,8S)-Dihydrodehydrodiconiferyl alcohol	*S. suberifolius*	[40]
**71**	(−)-(7R,8S)-Dihydrodehydrodiconiferyl alcohol 4-O-*β*-Dglucopyranoside	*S. suberifolius*	[40]
**72**	(−)-(7S,8R)-Dihydrodehydrodiconiferylalcohol 4-O-*β*-D-glucopyranoside	*S. suberifolius*	[40]
**73**	(+)-(7S,8R)-Dihydrodehydrodiconiferyl alcohol	*S. suberifolius*	[40]
**74**	(+)-(7S,8R)-Erythro-4,7,9,9′-tetrahydroxy-3,3′-dimethoxy-8-O-4′-neolignan	*S. suberifolius*	[40]
**75**	(−)-Symplocosneolignan A	*S. suberifolius*	[40]
**76**	(−)-7-O-Ethylguaiacylglycerol (15)	*S. suberifolius*	[40]
**77**	2[4-(3-Hydroxypropyl)-2-methoxyphenoxy]-1,3-propanediol	*S. suberifolius*	[40]
**78**	Dihydroconiferyl alcohol	*S. suberifolius*	[40]
**79**	3,3-Bis(3,4-dihydro-6-methoxy-2H-1-benzopyran	*S. tonkinensis*	[41]
**80**	Rac-(8*α*,8′*β*)-4,4′-dihydroxy3,3′-dimethoxylignan-9,9′-diyldiacetate	*S. tonkinensis*	[41]
**81**	(–)-Secoisolariciresino	*S. tonkinensis*	[41]
**82**	4,4′-Dihydroxy-3,3′dimethoxy-9-ethoxy-9,9′-epoxylignan	*S. tonkinensis*	[41]
**83**	(2S,3R,4R)-4-[1-Ethoxy-1-(4-hydroxy-3-methoxy)phenyl]methyl-2(4-hydroxy-3-methoxy)phenyl-3-hydroxymethyl-tetrahydrofuran	*S. tonkinensis*	[41]
**84**	(–)-Neo-olivil-(9-O-9″)-seco-isolariciresinol	*S. tonkinensis*	[41]
**85**	Isolariciresinol	*S. tonkinensis*	[41]
**86**	Stytonkinol A	*S. tonkinensis*	[42]
**87**	Stytonkinol B	*S. tonkinensis*	[42]
**88**	Styraxin	*S. argentifolius*	[43]
**89**	Vladinol D	*S. argentifolius*	[43]
**90**	5-Carboxy7-methoxy-2-(3′,4′-methylenedioxyphenyl)benzofuran	*S. argentifolius*	[43]
**91**	5-((E)-2-Carboxyvinyl)-7-methoxy-2-(3′,4′-methylenedioxyphenyl)benzofuran	*S. argentifolius*	[43]
**92**	(–)-Machicendiol	*S. argentifolius*	[43]
**93**	Machicendona	*S. argentifolius*	[43]
**Terpenoids**			
**94**	Taraxerol	*S. japonica*	[7,45]
**95**	Oleanolic aldehyde acetate	*S. japonica*	[22,54]
**96**	Erythrodiol-3-acetate	*S. japonica*	[22,54]
**97**	Euphorginol	*S. japonica*	[22,54]
**98**	3*β*-Acetoxyolean-12-en-28-acid	*S. japonica*	[45]
**99**	3*β*-Acetoxy-17*β*-hydroxy-28-norolean-12-ene	*S. japonica*	[45]
**100**	Anhydrosophoradiol-3-acetate	*S. japonica*	[22,54]
**101**	Styraxosides B	*S. japonica*	[44]
**102**	Camellenodiol	*S. japonica*	[44]
**103**	Jegosaponins A	*S. japonica*	[46]
**104**	Jegosaponins B	*S. japonica*	[46]
**105**	Jegosaponins C	*S. japonica*	[46]
**106**	Jegosaponins D	*S. japonica*	[46]
**107**	3*β*-Acetoxy-28-hydroxyolean-12-ene	*S. japonica*	[47]
**108**	3*β*-Acetoxyolean-12-en-28-acid	*S. japonica*	[47]
**109**	3*β*-Acetoxyolean-12-en-28-aldehyde	*S. japonica*	[47]
**110**	3*β*-Acetoxy-17*β*-hydroxy-28-norolean-12-ene	*S. japonica*	[47]
**111**	6*β*-Hydroxy-3-*oxo*-11*α*,12*α*-epoxyolean- 28,13*β*-olide	*S. tonkinensis*	[4]
**112**	3*β*,6*β*-Dihydroxy-11*α*,12*α*-epoxyolean-28,13*β*-olide	*S. tonkinensis*	[4]
**113**	3*β*,6*β*-Dihydroxy-11-*oxo*-olean-12-en-28-oic acid	*S. tonkinensis*	[4]
**114**	3*β*-Hydroxy-12-*oxo*-13HR-olean-28,19*β*-olide	*S. tonkinensis*	[4]
**115**	19*β*-Hydroxy-3-*oxo*-olean-12-en-28-oic acid	*S. tonkinensis*	[4]
**116**	6*β*-Hydroxy-3-*oxo*-olean-12-en-28-oic acid	*S. tonkinensis*	[4]
**117**	Sumaresinolic acid	*S. tonkinensis*	[4,51]
**118**	Siaresinolic acid	*S. tonkinensis*	[4,51]
**119**	Oleanolic acid	*S. tonkinensis*	[4,51]
**120**	21-Benzoylbarringtogenol C	*S. officinalis* L.	[4]
**121**	Styrax-saponin A	*S. officinalis* L.	[49]
**122**	Styrax-saponin B	*S. officinalis* L.	[49]
**123**	Styrax-saponin C	*S. officinalis* L.	[49]
**124**	Deacylsaponin	*S. officinalis* L.	[49]
**125**	Oleanonic acid	*S. tonkinensis*	[42,51]
**126**	Betulonic acid	*S. tonkinensis*	[42,51]
**127**	Corosolic acid	*S. tonkinensis*	[42,51]
**128**	Maslinic acid	*S. tonkinensis*	[42,51]
**129**	Epibetulinic acid	*S. tonkinensis*	[42]
**130**	Betulinic acid	*S. tonkinensis*	[42,51]
**131**	2*α*,3*α*,24-trihydroxy-urs-12-en-28-oic acid	*S. argentifolius*	[43]
**Aromatic Compounds**			
**132**	*trans*-(Tetrahydro-2-(4-hydroxy-3-methoxyphenyl)-5-oxofuran-3-yl)methylbenzoate	*S. tonkinensis*	[52]
**133**	3-(4-Hydroxy-3-methoxyphenyl)-2-oxopropylbenzoate	*S. tonkinensis*	[52]
**134**	4-(*E*)-3-Ethoxyprop-1-enyl)-2-methoxyphenol	*S. tonkinensis*	[52]
**135**	Benzoic acid	*S. tonkinensis*	[52]
**136**	Vanillin	*S. tonkinensis*	[52]
**137**	Dehydrodivanillin	*S. tonkinensis*	[52]
**138**	Vanillic acid	*S. tonkinensis*	[52]
**139**	Coniferyl aldehyde	*S. tonkinensis*	[52]
**140**	Methylsyringin	*S. japonica*	[21]
**141**	Syringin	*S. japonica*	[7,21]
**142**	Isotachioside	*S. perkinsiae*	[39]
**143**	(2R,3R)-3,7,4′-Trihydroxy-5,3′-dimethoxyflavan 7-O-*β*-D-glucopyranoside	*S. suberifolius*	[40]
**144**	Methyl orsellinate	*S. suberifolius*	[40]
**145**	Ethyl orsellinate	*S. suberifolius*	[40]
**146**	Methyl *β*-orcinolcarboxylate	*S. suberifolius*	[40]
**Steroids**			
**147**	Stigmasterol	*S.* *macranthus*	[32]
**148**	Styraxosides A	*S. japonica*	[32]
**149**	Daucosterol	*S.* *macranthus*	[32]
**150**	*β*-Sitosterol	*S. perkinsiae*	[17]
**151**	(20R)-24Ethylcholest-5,22-dien-7-one	*S. argentifolius*	[43]
**Others**			
**152**	Kaempferol-3-*O*-(2″,4″-di-*O*-(*E*)-p-coumaroyl-*β*-D-glucopyranoside)	*S. pohlii*	[56]
		*S. camporum*	[56]
**153**	Kaempferol-3-*O*-(2″,6″-di-*O*-(*E*)-p-coumaroyl)-*β*-D-glucopyranoside	*S. pohlii*	[56]
		*S. camporum*	[56]
**154**	Quercetin	*S. pohlii*	[56]
		*S. camporum*	[57]
**155**	Kaempferol	*S. pohlii*	[56]
		*S. camporum*	[57]
**156** **157**	Koninginin T Koninginin U	*S. pohlii*	[57]
		*S. pohlii*	[57]
**158**	Jegosaponins A	*S. japonica*	[58]
**159**	Jegosaponins B	*S. japonica*	[58]

## Appendix C

**Table A3:** Structural information of the active molecule.

No.	Structure	Phytochemistry	Pharmacological Activity
**3**		*S. perkinsiae* *S. obassia*	MCF-7 (IC_50_ = 53.5 μg/mL) Hela (IC_50_ = 23.3 μg/mL) HL-60 (IC_50_ = 16.8 μg/mL)
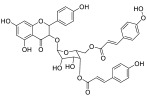
**4**		*S. perkinsiae* *S. japonica* *S. obassia* *S. agrestis* *S. ferrugineus* *S. pohlii* *S. camporum* *S. macranthus* *S. officinalis* *S. argentifolius*	Hep-2 (IC_50_ = 3.6 μg/mL) C6 (IC_50_ = 3.2 μg/mL) KB (IC_50_ = 22.11 μg/mL) HepG2 (IC_50_ = 18.15 μg/mL) Lu (IC_50_ = 21.50 μg/mL) Hemolytic activity (IC_50_ = 33 μg/mL) *C. sphaerospermum* (MIC = 5 mg/mL) *C. albicans* (MIC = 10 μg/mL) *S. aureus* (MIC = 10 μg/mL) *ATCC 27853* and *ATCC 6305* (MIC = 400 mg/mL) COX-1 (inhibition rate = 35.7% at 30 μM) Reduce doxorubicin- and methanesulfonate-induced DNA and chromosomal damage
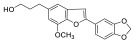
**5**		*S. perkinsiae* *S. japonica* *S. suberifolius* *S. macranthus* *S. obassia*	MCF-7 (IC_50_ = 27.9 μg/mL) Hela (IC_50_ = 23.3 μg/mL) *S. aureus* (MIC = 20 μg/mL) *C. albicans* (MIC = 15 μg/mL) HL-60 (IC_50_ = 47.8 μg/mL)
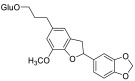
**6**		*S. perkinsiae* *S. macranthus* *S. obassia* *S. pohlii*	Estrogen biosynthesis
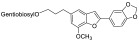
**7**		*S. perkinsiae* *S. macranthus*	Estrogen biosynthesis
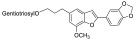
**10**		*S. perkinsiae*	Hemolytic activity (IC_50_ = 166 μg/mL)
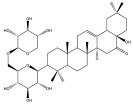
**11**		*S. perkinsiae*	MCF-7 (IC_50_ = 5.5 μg/mL) MDA-MB-231 (IC_50_ = 3.81 μg/mL)
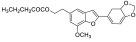
**14**		*S. perkinsiae* *S. japonica*	MCF-7 (IC_50_ = 15.0 μg/mL) MDA-MB-231 (IC_50_ = 13.71 μg/mL)
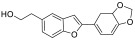
**17**		*S. ferrugineus* *S. camporum* *S. pohlii*	HeLa (IC_50_ = 5.3 μg/mL) C6 (IC_50_ = 4.9 μg/mL) *C. sphaerospermum* (MIC = 10 μg/mL) *C. albicans* (MIC = 12 μg/mL) *S. aureus* (MIC = 10 μg/mL) Th2 cytokines, iNOS, MMP-9 Reduce doxorubicin- and methanesulfonate-induced DNA and chromosomal damage
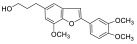
**18**		*S. ferrugineus* *S. pohlii*	*S. aureus* (MIC = 20 μg/mL) *C. albicans* (MIC = 20 μg/mL)
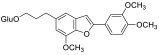
**19**		*S. ferrugineus*	*S. aureus* (MIC = 20 μg/mL) *C. albicans* (MIC = 15 μg/mL)
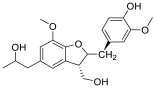
**26**		*S. japonica*	MMP-1 (inhibition rate = 62.1% at 10 μM)
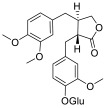
**28**		*S. japonica*	Hemolytic activity (IC_50_ = 123 μg/mL)
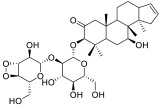
**30**		*S. japonica*	DPPH (IC_50_ = 380 μM)
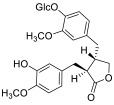
**31**		*S. japonica*	DPPH (IC_50_ = 278 μM)
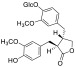
**32**		*S. japonica*	DPPH (IC_50_ = 194 μM)
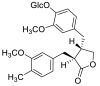
**33**		*S. japonica*	DPPH (IC_50_ = 260 μM)
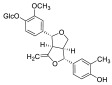
**50**		*S. agrestis*	EeAChE (IC_50_ = 1.4 μg/mL) hAChE (IC_50_ = 1.7 μg/mL)
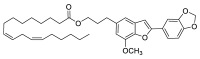
**51**		*S. agrestis*	EeAChE (IC_50_ = 2.0 μg/mL) hAChE (IC_50_ = 2.7 μg/mL)
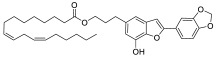
**52**		*S. agrestis*	EeAChE (IC_50_ = 1.4 μg/mL) hAChE (IC_50_ = 1.8 μg/mL)
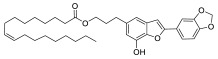
**53**		*S. agrestis*	EeAChE (IC_50_ = 2.2 μg/mL) hAChE (IC_50_ = 3.1 μg/mL)
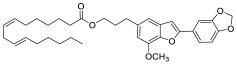
**86**		*S. tonkinensis*	Hela (IC_50_ = 26.75 μM) MCF-7 (IC_50_ = 45.16 μM)
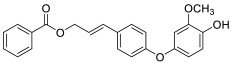
**87**		*S. tonkinensis*	MCF-7 (IC_50_ = 57.1 μM) Hemolytic activity (IC_50_ = 65 μg/mL)
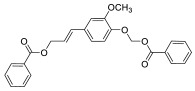
**92**		*S. argentifolius*	KB (IC_50_ = 96.01 μg/mL) HepG2 (IC_50_ = 86.60 μg/mL) Lu (IC_50_ = 106.86 μg/mL)
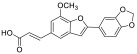
**95**		*S. japonica*	Human dermal fibroblasts (IC_50_ = 20 μM)
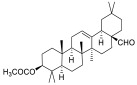
**96**		*S. japonica*	MMP-1 (inhibition rate = 73.1% at 0.01 μM)
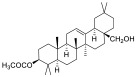
**100**		*S. japonica*	Human dermal fibroblasts (IC_50_ = 1.12 μM)
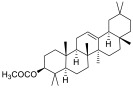
**101**		*S. tonkinensis*	Hemolytic activity (IC_50_ = 65 μg/mL)
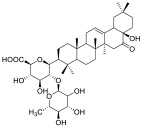
**108**		*S. japonica*	PTP1B (IC_50_ = 7.8 μg/mL)
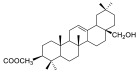
**109**		*S. japonica*	PTP1B (IC_50_ = 9.3 μg/mL)
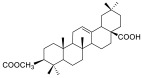
**112**		*S. tonkinensis*	HL-60 (IC_50_ = 27.5 μg/mL)
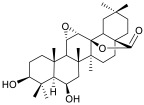
**116**		*S. tonkinensis*	HL-60 (IC_50_ = 14.2 μg/mL)
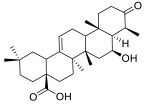
**118**		*S. tonkinensis*	HL-60 (IC_50_ = 29.0 μg/mL)
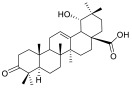
**119**		*S. tonkinensis*	HL-60 (IC_50_ = 8.9 μg/mL)
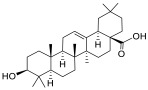
**125**		*S. tonkinensis*	hCES1A (IC_50_ = 0.49 mg/mL)
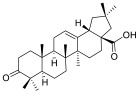
**126**		*S. tonkinensis*	hCES1A (IC_50_ = 1.48 μg/mL)
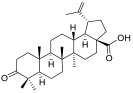
**129**		*S. tonkinensis*	hCES1A (IC_50_ = 0.041 μg/mL)
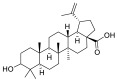
**144**		*S. suberifolius*	*A. solani* (inhibition rate = 58.41% at 100 μg/mL) *F. oxysporum* (inhibition rate = 67.39% at 100 μg/mL)
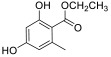
**145**		*S. suberifolius*	*A. solani* (inhibition rate = 59.31% at 100 μg/mL) *F. oxysporum* (inhibition rate = 45.65% at 100 μg/mL)
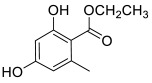
**146**		*S. suberifolius*	*F. oxysporum* (inhibition rate = 61.41% at 100 μg/mL) *P. cytospore* (inhibition rate = 86.72% at 100 μg/mL)
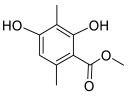
**149**		*S.* *macranthus*	iNOS, COX-2, TNF-a, and IL-1b
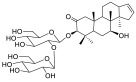
**152**		*S. pohlii* *S. camporum*	Separate coupled Schistosoma mansoni adult worms
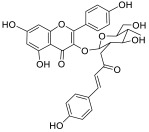
**155**		*S. pohlii* *S. camporum*	Separate coupled Schistosoma mansoni adult worms Kill adult schistosomes
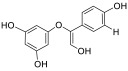
**156**		*S. pohlii*	AChE
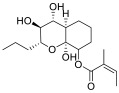
**158**		*S. japonica*	Hemolytic activity (IC_50_ = 2.1 μg/mL)
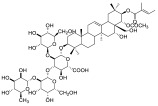
**159**		*S. japonica*	Hemolytic activity (IC_50_ = 20.2 μg/mL)
